# Hepatocellular carcinoma in patients cured of chronic hepatitis C: Minimal steatosis

**DOI:** 10.1002/cam4.5711

**Published:** 2023-04-20

**Authors:** Chiara Rocha, Erin H. Doyle, Chip A. Bowman, M‐Isabel Fiel, Ashley E. Stueck, Nicolas Goossens, Kian Bichoupan, Neal Patel, James F. Crismale, Jasnit Makkar, Sara Lewis, Ponni V. Perumalswami, Thomas D. Schiano, Yujin Hoshida, Myron Schwartz, Andrea D. Branch

**Affiliations:** ^1^ Department of Surgery—Transplant Division Icahn School of Medicine at Mount Sinai New York New York USA; ^2^ Division of Liver Diseases, Department of Medicine Icahn School of Medicine at Mount Sinai School New York New York USA; ^3^ Department of Medicine Icahn School of Medicine at Mount Sinai New York New York USA; ^4^ Department of Pathology Icahn School of Medicine at Mount Sinai New York New York USA; ^5^ Department of Pathology Dalhousie University Halifax Nova Scotia Canada; ^6^ Division of Liver Diseases, Department of Medicine Tisch Cancer Institute, Icahn School of Medicine at Mount Sinai New York New York USA; ^7^ Division of Liver Diseases, Department of Medicine Icahn School of Medicine at Mount Sinai New York New York USA; ^8^ Department of Radiology Columbia University New York New York USA; ^9^ Department of Radiology Icahn School of Medicine at Mount Sinai New York New York USA; ^10^ Department of Medicine University of Michigan Ann Arbor Michigan USA; ^11^ Department of Internal Medicine University of Texas Southwestern Medical Center Dallas Texas USA; ^12^ Department of Surgery Icahn School of Medicine at Mount Sinai New York New York USA; ^13^ Division of Gastroenterology Department of Medicine, Nuvance Health Danbury Hospital Danbury CT USA

**Keywords:** alpha‐fetoprotein, hepatitis C virus, hepatocellular carcinoma, sustained virological response

## Abstract

**Background:**

Successful treatment of hepatitis C reduces liver inflammation and fibrosis; however, patients remain at risk of developing hepatocellular carcinoma (HCC).

**Aims:**

To identify risk factors for new‐onset HCC in patients cured of hepatitis C.

**Methods:**

Imaging, histological, and clinical data on patients whose first HCC was diagnosed >12 months of post‐SVR were analyzed. Histology of 20 nontumor tissues was analyzed in a blinded manner using the Knodel/Ishak/HAI system for necroinflammation and fibrosis/cirrhosis stage and the Brunt system for steatosis/steatohepatitis. Factors associated with post‐SVR HCC were identified by comparison with HALT‐C participants who did not develop post‐SVR HCC.

**Results:**

Hepatocellular carcinoma was diagnosed in 54 patients (45 M/9F), a median of 6 years of post‐SVR [interquartile range (IQR) =1.4‐10y] at a median age of 61 years (IQR, 59–67). Approximately one‐third lacked cirrhosis, and only 11% had steatosis on imaging. The majority (60%) had no steatosis/steatohepatitis in histopathology. The median HAI score was 3 (1.25–4), indicating mild necroinflammation. In a multivariable logistic regression model, post‐SVR HCC was positively associated with non‐Caucasian race (*p* = 0.03), smoking (*p* = 0.03), age > 60 years at HCC diagnosis (*p* = 0.03), albumin<3.5 g/dL (*p* = 0.02), AST/ALT>1 (*p* = 0.05), and platelets <100 × 10^3^ cells/μL (*p* < 0.001). Alpha fetoprotein ≥4.75 ng/mL had 90% specificity and 71% sensitivity for HCC occurrence. Noncirrhotic patients had larger tumors (*p* = 0.002) and a higher prevalence of vascular invasion (*p* = 0.016) than cirrhotic patients.

**Conclusions:**

One‐third of patients with post‐SVR HCC did not have liver cirrhosis; most had no steatosis/steatohepatitis. Hepatocellular carcinomas were more advanced in noncirrhotic patients. Results support AFP as a promising marker of post‐SVR HCC risk.

## Graphical abstract

Hepatocellular carcinoma was diagnosed in 54 patients, and nearly one‐third did not have cirrhosis at the time of HCC diagnosis and the vast majority did not have liver steatosis/steatohepatitis on either imaging or histopathology. Non‐Caucasian race and advanced liver disease were risk factors for post‐SVR HCC; however, liver function was generally well‐preserved in the group without liver cirrhosis. Hepatocellular carcinomas were more advanced in noncirrhotic patients but otherwise had typical histopathologic and radiographic features.

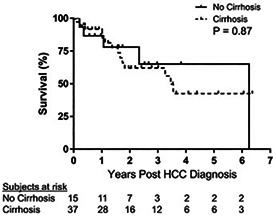

## 1 INTRODUCTION

Direct‐acting antiviral (DAA) drugs for chronic hepatitis C virus (HCV) infection have dramatically increased the number of patients who have achieved a sustained virological response (SVR) to HCV treatment. Although HCV cure reduces liver inflammation and fibrosis, fibrosis regression is often incomplete. In a paired‐biopsy study with 5 years of follow‐up, portal inflammation persisted in two‐thirds of patients post‐SVR.^1^ Patients remain at risk of developing hepatocellular carcinoma (HCC) after HCV cure.^2^, ^3^ The risk remains elevated for up to 10 years.^4^ Direct‐acting antiviral drugs allow nearly all patients to achieve an SVR, whereas interferon (IFN)‐based therapy had low SVR rates in patients with cirrhosis. The ability to cure HCV in patients with advanced liver disease is a great benefit; however, according to projections, over 50% of the HCV‐positive patients in the United States will have advanced fibrosis (F3) or cirrhosis (F4) by the time they achieve an SVR.^5^ In addition, patients who achieve an SVR in the future will be older than in the past due to aging of the baby boomer cohort, which constitutes the majority patients with current or past HCV infection in the United States.^6^ In the future, HCC risk after HCV cure will continue to be an important problem, and post‐SVR HCC surveillance will be an important tool to increase early detection.

Previous studies identified cirrhosis,^4^ male sex,^7^ older age,^8^, ^9^ lower platelets,^10^ and diabetes^11^ as baseline HCC risk factors in patients cured of HCV; however, a comprehensive analysis of imaging, histological, surgical, and clinical features of post‐SVR HCC has not yet been performed on patients in the United States who were cured of HCV during the era of IFN‐based therapy. The primary goal of this study was to conduct a detailed analysis of patients who had their first diagnosis of HCC more than a year after achieving an SVR and to determine the prevalence of liver cirrhosis and liver steatosis in patients developing HCC in the post‐SVR setting. A secondary goal was to identify factors associated with post‐SVR HCC.

## 2 METHODS

### 2.1 The Mount Sinai cohort of 54 case patients

The study group was comprised of 54 patients with an SVR to HCV therapy in whom HCC first was diagnosed more than 12 months of post‐SVR at the Icahn School of Medicine at Mount Sinai between January 2010 and April 2016. Patients were excluded if they had detectable HCV RNA in blood (indication of ongoing infection), had not received HCV treatment, had a history of HCC prior to SVR, or were diagnosed with HCC during HCV treatment or within the first 12 months of post‐SVR; these patients were excluded because they might have HCCs that had not yet acquired the imaging characteristics needed for a LI‐RADS 5 classification,^12^ as we^13^ and others^14^ previously reported in post‐SVR patients. Hepatocellular carcinoma was diagnosed via typical appearance on contrast‐enhanced CT or MRI,^15^ according to AASLD guidelines.^16^ At the time of HCC diagnosis, baseline data were collected on demographics, clinical laboratory values, comorbidities, social habits, imaging, and clinical staging of the tumor. Follow‐up data on HCC treatment and patient survival were extracted through June 2016. The study had approval of the Mount Sinai Institutional Review Board, GCO 10–0032, and was conducted as specified in the Helsinki Accord. A waiver of consent was granted.

Pathologic, imaging, or clinical findings were used to determine the presence or absence of cirrhosis. Briefly, histological data from nontumor tissue of biopsy and surgical specimens obtained within 12 months of the HCC diagnosis were used, when available (22 patients). Among the other patients, evidence of portal hypertension (esophageal varices and/or ascites) was used to establish the presence of cirrhosis. Radiographic findings suggestive of liver nodularity alone were not used to diagnose cirrhosis unless supported by additional clinical evidence of cirrhosis. If there was discordance between the clinical, laboratory, and radiographic data suggesting cirrhosis, the patient was classified as “indeterminate.” This occurred in two patients. They were excluded from the comparison of patients with and without cirrhosis.

### 2.2 Imaging technique

Imaging data from the time of HCC diagnosis were available for 49 patients (91%); the others did not have imaging data recorded at Mount Sinai. The patients underwent multiphase contrast‐enhanced MRI and CT examinations. MRI was performed at 1.5 T (*n* = 21) or 3 T (*n* = 4) using a variety of imaging platforms. Multiphase protocols consisted of arterial, portal venous, and late venous phases before and after the administration of gadolinium‐based contrast agents. CT scans were obtained using a variety of multidetector CT platforms. CT examinations included contrast‐enhanced imaging through the liver was performed, with hepatic arterial phase and portal venous phase imaging (60–70 s), after the initiation of a bolus of IV contrast material.

### 2.3 Image analysis

CT and MR images were reviewed on PACS (Centricity PACS, GE Healthcare) by two observers with 6 and 1 years of experience in abdominal imaging in consensus who were aware of the diagnosis of HCC. The images were reviewed for the presence of liver cirrhosis, steatosis, and evidence of portal hypertension (ascites, varices, and splenomegaly) using established criteria.^17^ Intrahepatic fat was detected on CT by previously described methods.^18^ Images were reviewed for the following: number of lesions, lesion size, lesion location, lesion margin, lesion enhancement characteristics, and presence of macrovascular invasion, which were defined as invasion involving the portal vein and hepatic vein branches. The dynamic enhancement characteristics of the lesions on contrast‐enhanced imaging were categorized as follows: typical wash‐in (during the arterial phase) and wash‐out (during the portal venous phase, equilibrium phase, or both phases), hypovascular, peripheral rim enhancement, and progressive whole lesion enhancement.

### 2.4 Histological examination of nontumor tissue

Twenty patients had surgical treatment of HCC at Mount Sinai yielding tissue that could be examined (two cases had tissue examined previously that was not available for further review). A blinded review of the nontumor portion of the specimens was conducted by an experienced liver pathologist, who analyzed the stage and grade of the hematoxylin/eosin (H&E)‐stained and trichrome‐stained tissue in parallel with 15 control slides of explants from patients with documented chronic HCV infection, but no HCC, at the time of transplantation. The modified Knodell/Ishak system was used to evaluate necroinflammation [histology activity index (HAI)] (scale, 0–18) and fibrosis stage (scale, 0–6)^19^; the Brunt system was used to score steatosis and steatohepatitis (scales, 0–3).^20^

### 2.5 Comparison with the HALT‐C cohort of post‐SVR patients who did not develop HCC

To identify factors associated with the development of new‐onset HCC after SVR, a Case–Control study was performed in which the Control group was comprised of post‐SVR subjects enrolled in the HALT‐C study who did not develop HCC during follow‐up.^21^ (NOTE: This is a separate Control group from the one in the histology study). The primary objective HALT‐C was to evaluate the effect of half‐dose pegylated‐interferon maintenance therapy in patients with chronic HCV infection; all patients had advanced fibrosis/cirrhosis at baseline. Prior to randomization (to maintenance therapy or observation), participants received antiviral treatment, allowing some to achieve an SVR. A follow‐up study of the SVR group (*n* = 180)^22^ was done a median of 7.1 years (IQR = 6.5–7.8) after they had been excluded from the postrandomization phase. Forty were lost to follow‐up or refused to participate, two developed HCC, and two died. The other 136 post‐SVR HALT‐C patients who had not developed HCC at the time of follow‐up comprised our Control group. This group was compared to 51 HCV‐monoinfected post‐SVR HCC Cases. Cases with HIV and/or HBV were excluded because co‐infection was an exclusion criterion in HALT‐C. An additional analysis was carried out on 89 HALT‐C patients who had both baseline and follow‐up measurements of AFP.

### 2.6 Statistical analysis

Characteristics of patients who were cirrhotic were compared with those who were not cirrhotic, using Fishers exact, Chi‐squared, and Mann–Whitney tests using the GraphPad Prism software (La Jolla, CA) and R version 3.2 (www.r‐project.org). Histological characteristics of cases were compared with those who had an active HCV infection at biopsy using Mann–Whitney tests using the GraphPad Prism software. Logistic regression was used to identify factors significantly associated with HCC in the Case–Control study. All variables with a *p*‐value <0.05 in univariable analysis were evaluated in multivariable modeling with stepwise variable selection based on Akaike information criteria (AIC). The paired Wilcoxon test was used to compare AFP levels pre‐ and post‐SVR. The survival rate was analyzed using Kaplan–Meier curves, and statistical significance was determined using the log‐rank test using GraphPad Prism. All *p*‐values <0.05 were considered statistically significant.

## 3 RESULTS

### 3.1 Description of the 54 cases

Characteristics of the 54 patients are shown in Table 1. The median age at the time of HCC diagnosis was 61 years (IQR, 59–67). The majority were male (83%). There were 28 Caucasians, 11 Hispanics, six Asians, seven African Americans, and two who did not declare race/ethnicity. Forty‐nine patients had imaging data available for review (CT, *n* = 14; MRI, *n* = 35), and 22 had histological data. Fifteen (28%) were classified as noncirrhotic and 37 (69%) as cirrhotic. Intrahepatic fat was detected in five of forty‐five patients (11%); assessment could not be performed in four patients. Twenty‐six of 37 HCCs in patients with cirrhosis were identified during routine surveillance.

**TABLE 1:** Baseline characteristics of the 54 Case patients with and without cirrhosis.

	All patients (*n* = 54)	No cirrhosis (*n* = 15)^a^	Cirrhosis (*n* = 37)^a^	*p*‐value
Male Sex^b^	45 (83%)	13 (87%)	30 (81%)	1.0^d^
BMI, kg/m^2^ ^c^	27.5 (24.6–30.3)	26.0 (22.7–28.3)	27.8 (24.4–31.2)	0.095^e^
HCV Treatment				0.30^d^
Peg‐IFN & RBV	49 (91%)	15 (100%)	32 (86%)	
Peg‐IFN, RBV, & SOF	1 (2%)	0	1 (3%)	
SOF & RBV	1 (2%)	0	1 (3%)	
SMV & SOF	2 (4%)	0	2 (6%)	
LDV, SOF, & RBV	1 (2%)	0	1 (3%)	
Age at HCC diagnosis, year^c^	61 (59–67)	61 (57–67)	61 (58–67)	0.83^e^
HCC diagnosed via Surveillance^b^	33 (61%)	6 (40%)	26 (70%)	0.061^d^
Underwent liver transplant^b^	6 (11%)	0	6 (16%)	0.16^d^
Natural MELD^c^	8 (6–11)	7 (6–11)	8.5 (7–13)	0.13^e^
HIV coinfected^b^	2 (4%)	1 (7%)	1 (3%)	0.50^d^
HBV coinfected^b^	2 (4%)	2 (13%)	0	**0.079** ^d^
Race^b^				0.12^f^
African American	7 (13%)	3 (20%)	3 (8%)	
Asian	6 (11%)	1 (7%)	5 (14%)	
Caucasian	28 (52%)	6 (40%)	21 (57%)	
Hispanic	11 (20%)	3 (20%)	8 (22%)	
Unknown	2 (4%)	2 (13%)	0	
Smoking history^b^	37 (69%)	11 (73%)	25 (68%)	0.75^d^
Alcohol history^b^	18 (33%)	4 (27%)	13 (35%)	0.75^d^
IVDU history^b^	11 (21%)	2 (13%)	9 (25%)	0.47^d^
Hypertension^b^	27 (50%)	5 (33%)	21 (57%)	0.22^d^
Diabetes^b^	16 (30%)	1 (7%)	14 (38%)	**0.040** ^d^
AST, IU/L^c^	32 (23–55)	26 (20–71)	33 (27–54)	0.28^e^
ALT, IU/L^c^	26 (21–39)	21 (17–65)	28 (22–38)	0.54^e^
AST/ALT^c^	1.22 (0.95–1.62)	1.25 (0.95–1.58)	1.23 (1.04–1.66)	0.62^e^
AFP, ng/mL^c^	12 (4–90)	15 (4–11,642)	7 (4–52)	0.43^e^
AFP > 10 ng/mL^b^	29 (54%)	9 (60%)	18 (49%)	0.55^d^
AFP >4.75 ng/mL^b^	38 (70%)	10 (67%)	26 (70%)	1.0^d^
Total Bilirubin, mg/dL^c^	0.7 (0.5–1.3)	0.7 (0.5–3.4)	0.8 (0.5–1.1)	0.80^e^
Albumin, g/dL^c^	4.2 (3.6–4.5)	4.4 (4.0–4.6)	4.0 (3.5–4.4)	**0.038** ^e^
Platelets × 10^3^ cells/μL^c^	147 (99–205)	212 (167–337)	107 (84–156)	**<0.0001** ^e^
Creatinine, mg/dL^c^	0.95 (0.84–1.10)	0.95 (0.82–1.04)	0.93 (0.86–1.12)	0.51^e^
INR^c^	1.1 (1.0–1.2)	1.0 (1.0–1.1)	1.1 (1.0–1.2)	**0.035** ^e^
Multiple Lesions on Imaging^b^	11 (20%)	2 (13%)	9 (24%)	0.48^e^
Liver Lobe involved^b^				0.50^f^
Right	34 (63%)	10 (67%)	23 (62%)	
Left	9 (17%)	4 (27%)	7 (19%)	
Bilateral	11 (20%)	1 (7%)	7 (19%)	
Tumor Size, cm^c^	2.5 (1.8–5.4)	7.4 (2.3–10.6)	2.2 (1.6–3.8)	**0.002** ^e^
Tumor Differentiation^b^				0.073^f^
Poor	6/22 (27%)	4/8 (50%)	2/14 (14%)	
Moderate	11/22 (50%)	4/8 (50%)	7/14 (50%)	
Well	5/22 (23%)	0/8	5/14 (36%)	
Within Milan Criteria^b^	35 (65%)	5 (33%)	29 (78%)	**0.004** ^d^
Vascular Invasion present^b^	27 (51%)	12 (80%)	15 (42%)	**0.016** ^d^
Intrahepatic fat on imaging^b^	5/45 (11%)	0/10	5/33 (15%)	0.32^d^

*Note*: Bolded values indicate statistical significance.

^a^ Excluded two patients (29 & 33) that had indeterminate cirrhosis diagnoses.

^b^ *n* (%).

^c^ Median (IQR).

^d^ Mann–Whitney test.

^e^ Fisher's exact test.

^f^ Chi‐squared test.

Over 75% of the patients presented with a single lesion on imaging; however, only 65% were within Milan criteria and only 41% were eligible for surgical resection.^23^ Forty‐six (94%) had discrete lesions, while three had diffuse infiltrative lesions. In most patients (36 of 46, 78%), the lesions had a characteristic wash‐in/wash‐out enhancement pattern on imaging. The tumor was poorly differentiated in six of 22 (27%) of the cases with biopsies performed.

Regarding clinical history, 50% of patients had hypertension and 30% had diabetes. Regarding social habits, most patients (69%) had a history of smoking tobacco, while 33% reported alcohol use and 21% IV drug use. One was co‐infected with HIV, one with HBV, and one with both HIV and HBV. The year of HCV treatment and SVR was known for 38 patients. Among them, HCC was diagnosed a median of 6 years of post‐SVR (IQR, 1.4–10). Most (91%) achieved a SVR using interferon‐containing regimens.

### 3.2 Comparison of HCC patients with and without liver cirrhosis

**FIGURE 1:**
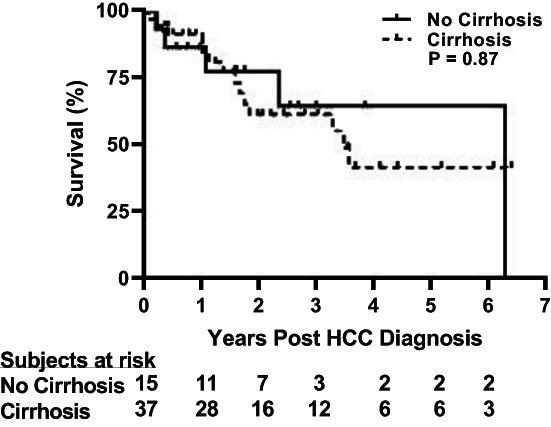
Survival curve of 52 patients who were diagnosed with HCC who had cirrhosis (dotted line, *n* = 37) or who did not have cirrhosis (solid line, *n* = 15). Analyzed using Kaplan–Meier curves and the log‐rank test.

Thirty‐seven patients with cirrhosis and 15 without cirrhosis were compared with each other. Patients without cirrhosis were less likely to have a history of diabetes, and they had lower INR, and higher albumin and platelet counts than patients with cirrhosis (Table 1). They had larger tumors (*p* = 0.002) and a higher prevalence of vascular invasion (*p* = 0.016). At a median follow‐up after HCC diagnosis of 581 days (IQR, 148–1089), survival of patients with and without cirrhosis was similar (Figure 1). Six cirrhotic patients received a liver transplant during that time.

### 3.3 Liver histopathology

Nontumor specimens from 20 cases were available for blinded histological analysis (Table 2); two additional cases had a biopsy performed and read at Mount Sinai, but tissue was not available for scientific review. Twelve (60%) showed cirrhosis (stage 5–6) according to the Knodell/Ishak classification system and eight (40%) did not. The median total HAI score was 3 points (IQR, 1.25–4), indicating that most had mild inflammation. Twelve (60%) had no steatosis and/or steatohepatitis. Interface hepatitis was present in 11 (55%), and portal inflammation was present in 16 (80%). These specimens were compared with those of 15 explants from patients with chronic HCV infection and no HCC. Compared with explants of patients with chronic HCV infection, the specimens of post‐SVR HCC liver had lower total HAI scores (*p* < 0.01) and lower fibrosis stage (*p* < 0.01) (Table 2).

**TABLE 2:** Histological features of the nontumor liver tissue of 20 Case patients compared with explants of 15 patients with no HCC and chronic HCV.

HCC post‐SVR cases *n* = 20	Cirrhosis present	Total Ishak/Knodell HAI score (0–18)	Components of Total Ishak/Knodell HAI Score				Architectural changes, fibrosis, cirrhosis (0–6)	Brunt steatosis grade (0–3)	Brunt Steato‐hepatitis grade (0–3)
			Periportal/ Periseptal Interface hepatitis (0–4)	Confluent necrosis (0–6)	Focal lytic necrosis, apoptosis, inflammation (0–4)	Portal inflammation (0–4)			
48	No	2	0	0	1	1	0	1	1
36	No	2	0	0	1	1	1	0	0
2	No	0.5	0	0	0/1	0	1	0	0
7	No	4	2	0	1	1	2	0	0
39	No	1	0	0	0	1	2	0	0
1	No	4	1	0	2	1	3	1	0
5	No	8	3	0	3	2	3	0	0
43	No	4	1	0	2	1	3	0	0
6	Yes	3	1	0	1	1	5	0	0
11	Yes	4	1	0	2	1	5	0	1
3	Yes	6	2	0	2	2	6	0	0
10	Yes	5	2	0	2	1	6	0	0
4	Yes	3	1	0	1	1	6	0	0
8	Yes	4	1	0	2	1	6	1	0
9	Yes	1	0	0	1	0	6	1	0
12	Yes	2	0	0	1	1	6	0	1
13	Yes	1	0	0	3	1	6	0	2
38	Yes	0	0	0	0	0	6	0	0
50	Yes	3	1	0	1	1	6	1	0
56	Yes	0	0	0	0	0	6	0	0

Group		Total Ishak/Knodell HAI score (0–18)	Components of Total Ishak/Knodell HAI Score				Architectural changes, fibrosis, cirrhosis (0–6)	Brunt steatosis grade (0–3)	Brunt steato‐hepatitis grade (0–3)
			Periportal/ periseptal interface hepatitis (0–4)	Confluent necrosis (0–6)	Focal lytic necrosis, apoptosis, inflammation (0–4)	Portal inflammation (0–4)			
HCC post‐SVR Cases^a^ *n* = 20		3 (1.25–4)	1 (0–1)	0 (0–0)	1 (1–2)	1 (1–1)	5.5 (2.25–6)	0 (0–0.8)	0 (0–0)
Non‐HCC cHCV Controls^a^ *n* = 15		6 (4.5–8)	2 (1–2.5)	0 (0–0)	2 (2–3)	2 (1–2)	6 (6–6)	0 (0–0)	0 (0–0)
*p*‐value^b^		0.0008	0.0099	NS	0.0017	0.0005	0.0016	0.057	0.724

^a^ Median (IQR).

^b^ Mann–Whitney test.

### 3.4 Comparison of MSSM patients who developed HCC post‐SVR and patients from the HALT‐C trial who achieved SVR and did not develop HCC

Univariable and multivariable logistic regression analyses were performed to identify factors associated with post‐SVR HCC. In these analyses, data from 51 Case patients who had had HCV mono‐infection prior to achieving an SVR were compared with data from 136 post‐SVR patients enrolled in the HALT‐C trial who had not developed HCC at a median follow‐up time of 7.1 years (IQR 6.4–7.8); Case patients with HIV or HBV infection were excluded. Laboratory values indicated that the post‐SVR/HCC group had a higher median MELD score, higher AST/ALT ratio, higher total bilirubin, lower albumin, and lower platelet counts, reflecting more advanced liver disease (Table 3). On multivariable analysis, six variables were significantly associated with HCC: non‐Caucasian race, a history of smoking, older age at HCC diagnosis, albumin level <3.5 mg/dL, platelet count <100 × 10^3^ cells/μL, and an AST/ALT >1 (Table 3). One‐third (31%) of the Case patients had only one or two of these six risk factors, indicating the need for additional biomarkers.

**TABLE 3:** Univariable and multivariable analysis of factors associated with HCC in a comparison of 51 Case patients and 136 no HCC, SVR patients in the HALT‐C study.

	Mount SInai HCC cohort (*n* = 51)	HALT‐C (*n* = 136)	Univariable			Multivariable		
			Odds ratio	95% Confidence interval	*p*‐value	Odds ratio	95% Confidence interval	*p*‐value
Male sex	42 (82%)	104 (76.5%)	0.7	0.3–1.7	0.434			
Caucasian	27/49 (55%)	108 (79%)	0.3	0.1–0.7	0.002	0.2	0.07–0.80	0.03
Smoking history	35 (69%)	49 (36%)	3.9	1.9–8.3	<0.001	6.0	2.0–21.0	0.003
Alcohol history	18 (35%)	46 (34%)	1.1	0.5–2.2	0.864			
Hypertension	26 (52%)	35 (26%)	3.1	1.5–6.5	0.001			
Diabetes	16 (32%)	19 (14%)	2.9	1.2–6.7	0.01			
BMI > 30 kg/m^2^	14 (28%)	44/93 (47%)	0.4	0.2–1.0	0.032			
Age > 60 years at time of HCC diagnosis (MSSM) or at follow up visit (HALT‐C)	29 (57%)	23 (17%)	6.4	3.0–14.0	<0.001	3.7	1.1–13.0	0.03
AST/ALT >1	37 (76%)	36/99 (36%)	5.3	2.4–12.7	<0.001	3.0	1.0–9.6	0.05
Albumin <3.5 g/dL	10 (20%)	2/101 (2%)	11.9	2.4–116.1	<0.001	26.0	2.4–775.0	0.02
Total Bilirubin >1 mg/dL	17 (33%)	13/101 (13%)	3.4	1.4–8.4	0.005			
Platelets <100 × 10^3^ cells/μL	15 (29%)	2/99 (2%)	19.8	4.3–186.4	<0.001	21.0	4.2–166.0	<0.001

**FIGURE 2:**
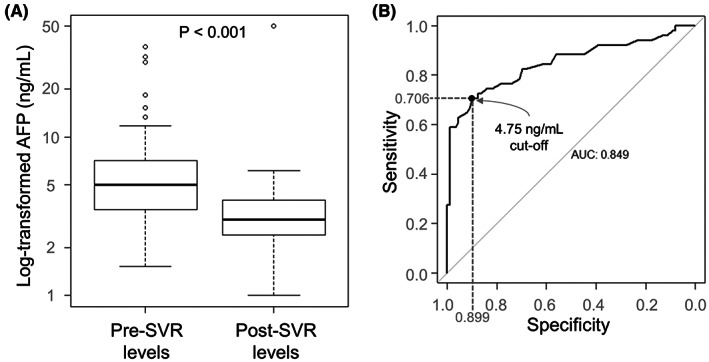
Decrease in AFP in patients cured of HCV. (A) AFP values in 89 HALT‐C subjects pre‐ and post‐SVR after log transformation. Statistical significance was calculated using paired Wilcoxon test. (B) Receiver operating characteristic (ROC) curve of log_10_ AFP for HCC diagnosis in SVR patients with the calculated area under curve (AUC).

To find additional factors that might differentiate post‐SVR patients with and without HCC, AFP levels were investigated. Among the 89 HALT‐C patients with available data, median pre‐ and post‐SVR AFP levels differed significantly and were lower in the post‐SVR specimens: 5.0 ng/mL (IQR, 3.5–7.1) versus 3.0 ng/mL (IQR, 2.4–4.0), *p* < 0.001. (Figure 2A). Because AFP levels decrease in patients cured of HCV,^24^, ^25^ they may be a more useful surveillance and early detection modality in post‐SVR patients than in patients with chronic HCV infection. To assess a potential diagnostic cutoff for HCC diagnosis in SVR subjects, we combined SVR HALT‐C Controls without HCC and our SVR Cases with HCC and assessed the diagnostic performance of AFP in distinguishing them from each other. We found that an AFP cutoff of 4.75 had 90% specificity and 71% sensitivity for distinguishing post‐SVR patients with and without HCC (Figure 2B).

## 4 DISCUSSION

The principal findings of this study are that almost one‐third (28%) of patients with HCC diagnosed for the first time ≥ 12 months after achieving an SVR were noncirrhotic at the time of diagnosis, and the majority (65%–88%) did not have steatosis or steatohepatitis. However, the uninvolved liver tissue showed portal inflammation in 80% of cases, indicating that inflammation was ongoing, consistent with prior data showing persistent portal inflammation in 66% of post‐SVR biopsies.^1^ Moreover, HCC in the noncirrhotic patients had features of more aggressive disease, including larger tumor size, a higher percentage of poorly differentiated tumors, presentation outside Milan criteria, and more frequent macrovascular invasion. Despite these adverse tumor characteristics, survival was similar in patients with and without cirrhosis. Our findings accord with published data. Gawrieh et al reported that patients with noncirrhotic HCC had larger tumors (8.9 vs. 5.3 cm) and were less frequently within Milan criteria (15% vs 39%).^26^ Schutte et al reported that patients without cirrhosis had more advanced tumor stage^27^ and Albeidawi et al reported that patients with HCV‐associated HCC without cirrhosis had larger tumors and more macrovascular invasion than patients with cirrhosis.^28^ A recent comparison of HCC in 30 noncirrhotic and 20 cirrhotic livers in patients with nonalcoholic steatohepatitis revealed that HCC in noncirrhotic liver had a higher mutational burden and was more likely to have satellite lesions and vascular invasion than HCC in cirrhotic liver.^29^ The worse tumor characteristics in patients without liver cirrhosis could reflect inherent differences in tumor biology and/or the consequences of late diagnosis. Most patients with well‐preserved liver function and noncirrhotic liver do not meet current AASLD guideline criteria for HCC surveillance,^16^ which could result in diagnosis at a late, symptomatic stage. There is an urgent need for new biomarkers of HCC that could make HCC surveillance more feasible and accessible.

One such potentially actionable biomarker for the presence of HCC in post‐SVR patients is AFP, which is supported by the results of our study. In patients with chronic HCV infection, AFP may be elevated in the absence of HCC, especially in patients with advanced fibrosis or cirrhosis. This background elevation reduces its utility as a surveillance tool, which is reflected in published sensitivity and specificity of 61% and 81%, respectively.^30^ As these test parameters are generally inadequate for an effective surveillance test, higher sensitivity modalities such as imaging with ultrasound, CT, and MRI are used instead.^31^ However, the background elevation of AFP is diminished by HCV cure, as was previously demonstrated.^32^ As such, AFP measurements may have a much greater power to identify patients with HCC after they have been cured of HCV. Our measurements of AFP post‐SVR are consistent with published data.^25^, ^33^, ^34^ Alpha fetoprotein may emerge as a useful screening modality for HCC in post‐SVR patients and should be investigated for this purpose, both alone and in combination with current and novel imaging methods^35^ and emerging serum biomarkers.^36^ Given current technologies, patients without cirrhosis who do not meet AASLD criteria for HCC surveillance, which entails abdominal ultrasound every 6 months, might benefit from serial AFP measurement.

Another way to improve HCC surveillance would be to identify prognostic factors, in addition cirrhosis that could be used to identify high‐risk patients, enabling their enrollment in surveillance programs. The development of HCC risk calculators is an area of active research. Post‐SVR AFP levels are promising components of HCC risk indices. In a multivariable model, among patients cured of HCV with IFN‐based regimens, a post‐SVR AFP >5 ng/mL had a hazard ratio of 8.1 (95% CI = 2.7–23.9).^37^ Similarly, in a multivariable model, among noncirrhotic patients cured of HCV with DAAs, a post‐SVR AFP >5 ng/mL had a hazard ratio of 4.9 (5% CI = 1.9–12.4).^38^ We found six additional variables that might be tested as components of a composite risk score: non‐Caucasian race, a history of smoking, older age, low albumin, low platelets, and AST/ALT >1. We^39^ and others^40^, ^41^, ^42^, ^43^, ^44^, ^45^ previously reported that African Americans have better liver function and less liver damage at the time of HCC diagnosis than other patients. These findings highlight the need for special vigilance in African American patients with chronic liver diseases, especially those with a history of smoking.

Several characteristics of patients with post‐SVR HCC that we identified (older age and indicators of more advanced liver disease) have been reported previously.^2^, ^3^, ^46^ Among these characteristics, age and advanced liver disease are particularly noteworthy for public health planning. Post‐SVR patients over 65 years of age have a 6.5‐fold higher annual incidence of HCC than post‐SVR patients under 65.^47^ Post‐SVR HCC will continue to be a significant problem in the years ahead as the baby boomer cohort ages and DAAs allow patients with advanced liver disease to achieve an SVR.^48^

The multidisciplinary approach we used is a major strength of the investigation. We provided detailed information on post‐SVR HCC, including CT and MRI imaging, histology, clinical outcomes, and surgical findings. Limitations of the study include the lack of a validation cohort, the small sample size, and the lack of complete and prospectively collected data, which occurred because many patients were referred to our tertiary center for HCC treatment after having received HCV treatment elsewhere, limiting data about HCV genotype, viral load, and HCV treatment regimen. Data about fibrosis stage prior to the HCC diagnosis were also not available and therefore we were not able to determine whether the patients without cirrhosis had cirrhosis in the past. However, we suspect that cirrhosis had been present and regressed in some patients because HCC was detected via surveillance in 40% of patients without cirrhosis confirmed at imaging and/or pathology. Because of the limitations of our study, our findings should be interpreted with caution; however, despite the limitations, our findings establish a solid foundation for future prospective investigations by highlighting features of post‐SVR HCC that may impact future surveillance strategies.

Almost one‐third of patients did not have cirrhosis at the time of HCC diagnosis and the great majority did not have liver steatosis or steatohepatitis. A better understanding of etiologic factors for HCC in post‐SVR patients is needed to allow screening tools to be developed and targeted to those most likely to benefit. Serial AFP measurements merit investigation as components of surveillance strategies for patients who have cleared HCV.

## AUTHOR CONTRIBUTIONS

**Chiara Rocha:** Conceptualization (equal); resources (equal). **Erin Doyle:** Conceptualization (equal); writing – original draft (equal). **Chip A Bowman:** Writing – review and editing (equal). **Maria Isabel Fiel:** Data curation (equal); resources (equal). **Ashley Stueck:** Investigation (equal); methodology (equal); resources (equal). **Nicolas Goossens:** Investigation (equal); methodology (equal); validation (equal). **Kian Bichoupan:** Investigation (equal). **Neal Patel:** Investigation (equal). **James Crismale:** Investigation (equal); validation (equal). **Jasnit Makkar:** Formal analysis (equal); investigation (equal). **Sara C Lewis:** Data curation (equal); formal analysis (equal); investigation (equal); resources (equal); software (equal). **Ponni Perumalswami:** Investigation (equal). **Thomas Schiano:** Investigation (equal); methodology (equal); writing – original draft (equal); writing – review and editing (equal). **Yujin Hoshida:** Investigation (equal); methodology (equal). **Myron Schwartz:** Investigation (equal); methodology (equal); writing – review and editing (equal). **Andrea D Branch:** Conceptualization (equal); formal analysis (equal); funding acquisition (equal); investigation (equal); methodology (equal); project administration (equal); resources (equal); writing – original draft (equal); writing – review and editing (equal).

## FUNDING INFORMATION

Supported by NIH (ADB & YH), NIDDK (ADB & YH), NIDA (ADB), FLAGS foundation (YH), Nuovo‐Soldati Cancer Research Foundation (YH), and Irma T. Hirschl Trust (YH). DA031095 and DK090317 (ADB). Advanced training grant from Geneva University Hospital (NG). DK099558 (YH). Virus–host interactions training grant from Icahn School of Medicine at Mount Sinai (EHD).

## Data Availability

Data sharing is not applicable to this article as no new data were created or analyzed in this study.
